# Mapping and Introgression of QTL Involved in Fruit Shape Transgressive Segregation into ‘Piel de Sapo’ Melon (*Cucucumis melo* L.)

**DOI:** 10.1371/journal.pone.0104188

**Published:** 2014-08-15

**Authors:** Aurora Díaz, Belkacem Zarouri, Mohamed Fergany, Iban Eduardo, José M. Álvarez, Belén Picó, Antonio J. Monforte

**Affiliations:** 1 Instituto de Biología Molecular y Celular de Plantas (IBMCP). Universidad Politécnica de Valencia (UPV)-Consejo Superior de Investigaciones Científicas (CSIC), Valencia, Spain; 2 Laboratorio de Biología Molecular. Dpto. Investigación Agroalimentaria. Instituto Madrileño de Investigación y Desarrollo Rural, Agrario y Alimentario (IMIDRA), Alcalá de Henares, Madrid, Spain; 3 Centre de Recerca en Agrigenòmica (CRAG), IRTA-CSIC-UAB, Bellaterra, Cerdanyola del Vallès, Barcelona, Spain; 4 Centro de Investigación y Tecnología Agroalimentaria de Aragón (CITA), Zaragoza, Spain; 5 COMAV-UPV, Institute for the Conservation and Breeding of Agricultural Biodiversity, Universitad Politécnica de Valencia, Valencia, Spain; Pennsylvania State University, United States of America

## Abstract

A mapping F_2_ population from the cross ‘Piel de Sapo’ × PI124112 was selectively genotyped to study the genetic control of morphological fruit traits by QTL (Quantitative Trait Loci) analysis. Ten QTL were identified, five for FL (Fruit Length), two for FD (Fruit Diameter) and three for FS (Fruit Shape). At least one robust QTL per character was found, *flqs8.1* (LOD = 16.85, R^2^ = 34%), *fdqs12.1* (LOD = 3.47, R^2^ = 11%) and *fsqs8.1* (LOD = 14.85, R^2^ = 41%). *flqs2.1* and *fsqs2.1* cosegregate with gene *a* (andromonoecious), responsible for flower sex determination and with pleiotropic effects on FS. They display a positive additive effect (*a*) value, so the PI124112 allele causes an increase in FL and FS, producing more elongated fruits. Conversely, the negative *a* value for *flqs8.1* and *fsqs8.1* indicates a decrease in FL and FS, what results in rounder fruits, even if PI124112 produces very elongated melons. This is explained by a significant epistatic interaction between *fsqs2.1* and *fsqs8.1*, where the effects of the alleles at locus *a* are attenuated by the additive PI124112 allele at *fsqs8.1*. Roundest fruits are produced by homozygous for PI124112 at *fsqs8.1* that do not carry any dominant A allele at locus *a* (PiPiaa). A significant interaction between *fsqs8.1* and *fsqs12.1* was also detected, with the alleles at *fsqs12.1* producing more elongated fruits. *fsqs8.1* seems to be allelic to QTL discovered in other populations where the exotic alleles produce elongated fruits. This model has been validated in assays with backcross lines along 3 years and ultimately obtaining a *fsqs8.1*-NIL (Near Isogenic Line) in ‘Piel de Sapo’ background which yields round melons.

## Introduction

Melon (*Cucumis melo* L., 2n = 2x = 24) is one of the most important horticultural crops, ranking in the ninth position of the world production of vegetables, with more than 27 million tons in 2012 [1]. In the last two decades, melon production has experienced a 29% rise, what has been accompanied by an increase of 8% in the area harvested. It does not seem feasible to achieve the same gain in production in the next decades by increasing the land dedicated to its cultivation, so the efforts of plant breeders should be directed to improve traits having a great potential to increase yield and fruit quality. In this sense, fruit morphology can be considered a quality trait as it is one of the first features to be perceived. Although consumers associate particular sizes and forms to certain market classes (i.e. medium size and round cantaloupes or large and oval inodorus), a whole world of possibilities is being opened up before us with the development of products with original appearance, either by traditional breeding, like the ZESPRI GOLD kiwifruit cultivar [2], or by genetic engineering, like the purple tomato [3]. Modern market trends demand smaller melons, as the family size is progressively falling; and with rounder shapes, what minimizes costs and mechanical damages during storage and transport.

Melon germplasm shows an impressive array of fruit sizes and morphologies [4]–[6], ranging from a few tens of grams up to 10 Kg and from slightly flat to extremely elongated shapes. Most of this diversity is found in traditional Central Asian landraces and wild melons [7]–[8]. This gene pool is considered “exotic” when compared to the popular Occidental varieties (i. e. Galias, honeydews, western shippers, cantaloups, and inodorus like ‘Piel de Sapo’, from here on referred to as ‘PS’) although it represents a valuable source of variability with a huge potential in breeding programs. In this sense, the accession PI124112 (*C. melo* ssp. *agrestis* var. *momordica*) collected in 1937 in India is genetically very distant from commercial cultivars, as revealed by studies carried out with SSR (Simple Sequence Repeat) [5], [9], and AFPL (Amplified Fragment Length Polymorphism) and SNP (Single Nucleotide Polymorphism) [10] markers, and shows resistance to several fungal and viral diseases (reviewed in [7]).

A few major genes with pleiotropic effects on melon fruit shape (FS) are currently known, like *andromonoecious* (*a*), that has been recently cloned [11] and is responsible for sex determination together with *gynoecious* (*g*), and like *pentamerous* (*p*), that controls carpel number. However, most of the phenotypic variation observed in FS seems to be caused by other Quantitative Trait Loci (QTL), as demonstrated by a number of QTL mapping studies that have been undertaken to genetically dissect its molecular basis [12]–[18].

Genotyping technologies have experienced an authentic revolution in the last few years [19]–[20], what has led to a renewed interest in QTL mapping. The availability of affordable high-throughput genotyping platforms will enable researchers to annotate more precisely the genomic regions of interest and speed up drastically the formerly time-consuming process of obtaining valuable plant material. All this will make possible to validate QTL and study putative interactions among them, at the same time that favorable alleles are transferred into elite varieties with a minimal drag of non-targeted fragments with potential detrimental effects. Consequently, it is expected that in a near future, new QTL will be added to those already cloned in important crops, like the QTL for fruit sugar content *Brix9-2-5* [21], for fruit size *fw2.2* [22], and those for fruit shape *ovate* [23], *fasciated* [24] and *sun* [25] in tomato; a QTL for grain shattering *sh4* [26] and those for flowering date *Hd1* [27], *Hd6* [28], *Hd3a* [29], and *Ehd1* [30] in rice; and the QTL for plant architecture *Tb1* [31] and flowering time *Dwarf8* [32] and *Vgt1* [33] in maize, among others (reviewed in [34]–[35]). In most cases, the strategy followed was the positional cloning, for what the availability of Near-Isogenic Lines (NILs) was essential, highlighting the importance of generating the appropriate genetic material to be successful. In this sense, the development of a collection of NILs in melon between the Korean cultivar ‘Songwhan Charmi’ and ‘PS’ [36] has allowed to dissect in single Mendelian factors economically important traits as fruit morphology [14], [17], climacteric ripening [37] and virus resistance [38].

In the present work, we identify and localize several QTL associated with melon fruit morphological traits in a cross between the Spanish cultivar ‘PS’ and the Indian accession PI124112 and study the interaction between those with the strongest and most robust effects. Furthermore, we have validated the QTL for FS *fsqs8.1* in a QTL-NIL developed in the genetic background of the cultivar ‘PS’, what produces totally round melons instead of the typical oval ones.

## Materials and Methods

All experiments were conducted in compliance with the current Spanish laws. No specific permissions were required for the experiments carried out in these two locations, Cabrils and Zaragoza, as they are research institutes, Institut de Recerca i Tecnologia Agroalimentaria (IRTA) and Centro de Investigación y Tecnología Agroalimentaria de Aragón (CITA), respectively. Moreover, authors IE and JMA belong to IRTA and CITA, respectively.

### Plant material and phenotypic evaluation

**Figure 1:**
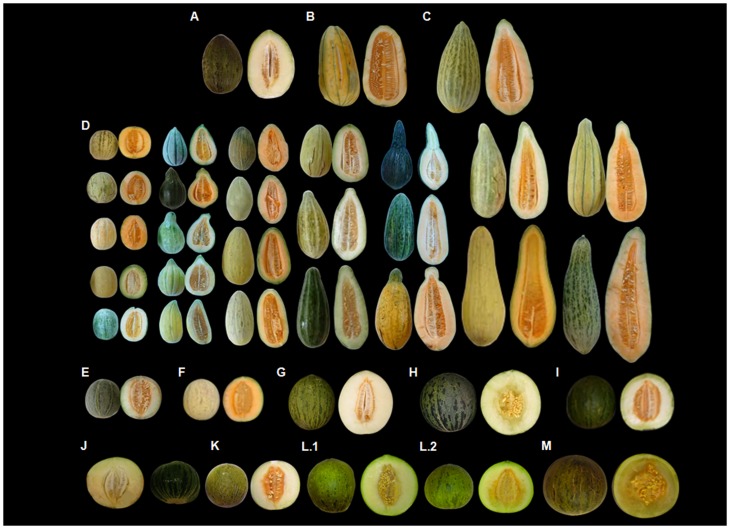
Fruit Shape (FS) polymorphism at each stage of the crossing program to obtain the *fsqs8.1*-NIL. Fruits of (A) ‘PS’; (B) PI124112; (C) 2M158-3 (F_1_); (D) different F_2_ plants to show the transgressive segregation of FS; (E) 3M70-47 (F_2_); (F) 5M113-1 × ‘PS’ (BC1); (G) 6M59-13 × ‘PS’ (BC2); (H) 7M36-1 × ‘PS’ (BC3); (I) 8M42-24 × ‘PS’ (BC4); (J) 9M7-15 ∶ (BC4S1); (K) 10M2-30 × ‘PS’ (BC5); (L) 11M27 ∶ (BC5S1), (L.1) 11M27-20: heterozygous at *fsqs8.1* and (L.2) 11M27-11: homozygous at *fsqs8.1*; (M) the *fsqs8.1*-NIL 12M57-3 OP (BC5S2).

The F_2_ plants of the mapping population were derived from a single cross between the monoecious Indian landrace accession PI124112 (*momordica* group) and the andromonoecious Spanish cultivar ‘PS’ (*inodorus* group) (Figure 1). PI124112 typically produces medium size, elongated and non-sweet fruits, whereas fruits coming from ‘PS’ are big, oval-shaped and very sweet [4]–[5].

A multi-location assay was conducted in summer of 2003 with a total of approximately 400 F_2_ plants. On one hand, 200 of them were randomly distributed in a greenhouse at IRTA in Cabrils (Barcelona, Spain), grown in drip-irrigated peat bags and self-pollinated by hand. The remaining 200 plants were randomized in an open field without irrigation at CITA, located in Zaragoza (Spain) and allowed to open-pollinate. The fertilization method became irrelevant as all the fruit except the seed content is derived from the maternal genitor. Ten plants of every genitor plus the F_1_ were included in both trials.

Fruit length (FL) and maximum fruit diameter (FD) were measured on longitudinal sections of collected fruits and then, FS was calculated as the ratio of FL over FD.

The absence of statistically significant genotype-by-environment (G×E) interactions was tested by calculating the least square means of FS in a two-way ANOVA with the data coming from six to nine replicates of F1, PI124112 and ‘PS’ in Cabrils and Zaragoza as a necessary condition to merge the data coming from the two locations.

Pair-wise Pearson's correlation coefficient was calculated between the three traits with the data coming from the whole F_2_ population and also with those obtained in the two location assays (Cabrils and Zaragoza) independently.

Percent mid-parental heterosis (MPH) was calculated using the data derived from a total of 15 replicates of each of the genotypes, F_1_ and the both parents, as:
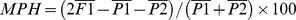



where 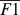, 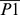 and 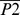 are the mean values of the F_1_, ‘PS’ and PI124112, respectively, for the trait under study.

One-way ANOVA and t-Student mean comparison tests were carried out with the data coming from the whole F_2_ population and also with those obtained in the two location assays independently.

All statistical analyses were performed using JMP v5.1.2 software for Windows (SAS Institute Inc., Cary, NC).

### DNA marker analysis in the mapping population

A selective genotyping strategy [39], which exploits maximum phenotypic differences with a minimal labor and time investment, was approached to study the FS trait. The plants selected to be genotyped were those with values more extreme than the mean of the F_2_ population ±1SD from each location, resulting in 84 individuals from both locations (File S1).

DNA was isolated from young leaves according to [40], with slight modifications and genotyped with a total of 133 markers (66 SSRs, 65 SNPs and 2 Cleaved Amplified Polymorphic Sites, CAPS) chosen to cover all the genome among those available in the literature (File S2). SSR markers were amplified following the protocol with M13-tailed forward primers plus dye labeled M13 primers described by [41] and subsequently analyzed on a 3130 Genetic Analyzer (Applied Biosystems). Genotyping with SNP markers was carried out using the Sequenom MassArray iPLEX system (Illumina), followed by MALDI-TOF mass spectrometry at Servicio de Investigaciones Biomédicas, Unidad Central de Investigación (University of Valencia, Valencia, Spain). Amplifications of the CAPS markers, AluICAPS [11] and PSI_25-H03 [42], were performed in 25-µl volume solutions containing 20 ng of genomic DNA, 7.5 mM Tris HCl (pH 9.0), 5 mM KCl, 2 mM (NH_4_)_2_SO_4_, 2.5 mM MgCl_2_, 0.2 mM of each dNTP, 0.2 µM of reverse and forward primers and 2.5 U of DNA polymerase (Biotools, Madrid, Spain). PCRs were carried out on a TC-512 thermocycler (Techne, UK) programmed with an initial denaturation step at 94°C for 5 min, followed by 35 cycles of 94°C for 30 s, 62°C for 45 s and 72°C for 1 min, plus a final elongation step at 72°C for 5 min. A 10-µl volume of the AluICAPS and PSI_25-H03 PCR products were digested with the restriction enzymes *Alu*I and *Bsh*1236I (Fermentas, Fisher Scientific, Madrid, Spain), respectively, following the supplier's recommendations and separated by agarose gel-electrophoresis.

### Linkage map construction and QTL analysis

The linkage map was constructed using MAPMAKER 3.0 [43] and the distances were calculated with the Kosambi function [44]. Markers were associated with the group command with LOD>3.0. When possible, they were put in order within each group using the order command with LOD>3.0, followed by a verification with the ripple command and the building of the framework map. If necessary, remaining markers in the group were included with the place command. If the previous approach did not render any map, the compare function was used to determine the best possible order for five starting markers, adding the remaining ones with the try command. In the case of groups with a low number of markers, the order and distances were estimated using the group command, followed by compare, what generated the framework map. Finally, maps were drawn with MapChart version 2.2 [45].

QTL analysis was performed for each location independently and for data from both locations merged together using Windows QTL Cartographer 2.5 [46] with the composite interval mapping (CIM, [47]) procedure. The LOD score threshold for a significant level p<0.05 was calculated by a permutation test with 1000 resamplings, giving a value of LOD>2.5 for the whole population for all traits and larger numbers in the individual populations. Additionally, the threshold LOD>2.5 was also established for the single location analyses. Markers associated to the most robust QTL (6 out of the 10 QTL as several of them share the same marker as the strongest linked one, i.e. *flqs8.1* and *fsqs8.1*) were genotyped in all F_2_ plants in order to obtain unbiased estimates of the gene action (d/[a]). The QTL were named according to the nomenclature system adopted by [48], where the first letters designate the trait abbreviation, followed by a “Q” that stands for QTL, then a letter referred to the mapping experiment (“s”, in this case), followed by a digit representing the linkage group (LG) to which the QTL maps, and then followed by a dot and a final digit that distinguishes different QTL from the same experiment on the same LG. In those cases in which two overlapping QTL were identified in a limited region and for the same trait, they were effectively considered as only one QTL.

### Study of QTL interactions

Epistatic interactions between the FS QTL detected were investigated by two-way ANOVA with the JMP v5.1.2 software for Windows and using the genotypic data from the linked markers in all F_2_ plants (not only the subset employed for the selective genotyping). These markers were AluICAPS in the case of *fsqs2.1*, CMAT141 for *fsqs8.1*, and ECM 67 for *fsqs12.1*. Interactions were considered to be statistically significant when *p*≤0.05. Sum of squares values were used to calculate the percentage of the phenotypic variation explained by the interaction between two QTL (R^2^).

### QTL validation by developing a QTL-NIL

The strategy followed to validate one of the FS QTL detected and to introgress it into the ‘PS’ elite cultivar consisted in selecting a single F_2_ plant from the trial located in Cabrils, that carried the PI124112 alleles at the QTL region and also showed round fruit, and backcross it to the parental ‘PS’ (Figure S1). Five rounds of backcrossing guided by a doubled selection, phenotypic (favoring round-shaped fruits) and assisted by markers linked to the target QTL (to monitor the introgressions harboring PI124112 alleles), were carried out. Simultaneously, background markers covering all the genome were also employed to gradually increase the percentage of the recurrent parent genome by selecting against the donor (PI124112) genome. An advanced backcross selection, 10M2-30, was genotyped with 548 SNPs using the Golden-Gate assay (Illumina, San Diego, CA) described in [10] in order to verify introgression size and integrity, and the composition of the genomic background. After two cycles of self-fertilization, the definitive introgression line in the BC5S2 generation was obtained (Figure 1M).

Phenotypical evaluations of the fruits were conducted in the greenhouse at several stages of the backcross scheme. In 2008, 42 fruits collected from 37 plants belonging to three full-siblings BC4 families (8M42-37, 41 and 42) carrying the PI124112 allele for the FS QTL in LG VIII (homozygous and heterozygous for two linked markers placed 23.3 cM apart in the genetic map) were evaluated in Cabrils. In 2009, BC4S1 plants (Figure S1) were genotyped with the markers linked to the QTL and 30 of their fruits were phenotyped in a greenhouse at Polytechnic University of Valencia. The cosegregation of markers and FS phenotypes was assessed by ANOVA. Finally, in 2012, melon fruits from the BC5S2 family 12M57 and the parental cultivar ‘PS’ were analyzed in Paiporta (Valencia). 12M57, derived from a homozygous plant for the closest markers to the FS QTL *fsqs8.1* (11M27-11, Figure S1), contained the introgression for the PI124112 allele at those same markers linked to the QTL. Means of the fruits produced by 10 12M57 plants were compared with means of the fruits collected from 15 ‘PS’ plants by a t-test.

## Results

### Phenotypic variation in morphological traits

Cultivar ‘PS’ produces oval-shaped fruits (FS = 1.4±0.1; Table 1 and Figure 1A), while PI124112 displays elongated fruits (FS = 1.7±0.2; Table 1 and Figure 1B). The F_1_ exhibited higher FS values (FS = 2.0±0.2, Table 1 and Figure 1C), in accordance with best-parent heterosis for this character previously observed in melon [16], particularly in hybrids between exotic accessions and ‘PS’ (*inodorus* group), caused mainly by an increase in FL [5]. No significant G×E interaction was observed for FS, therefore the parental mean values were calculated with the data coming from both locations. In Cabrils, the MPH values were 77%, 10% and 60% for FL, FD and FS, respectively; in the assay located in Zaragoza, 120%, 34% and 68%, respectively; and for the whole set of data, 100%, 24% and 64%, respectively. In all cases, heterosis was confirmed and, though there are slight differences in the MPH values for each character among assays, the ranking remains unaltered.

**Table 1:** Mean and Standard Deviation (SD) values of the traits for the parents, the F_1_ and the whole F_2_.

	FL		FD		FS	
	Mean	SD	Mean	SD	Mean	SD
‘PS’	19.5	1.3	14.0	1.1	1.4	0.1
PI124112	14.8	1.8	9.2	1.1	1.6	0.2
F_1_	25.1	3.5	12.7	1.8	2.0	0.2
F_2_	19.5	5.7	11.7	2.0	1.7	0.5

FL: Fruit Length; FD: Fruit Diameter; FS: Fruit Shape.

**Figure 2:**
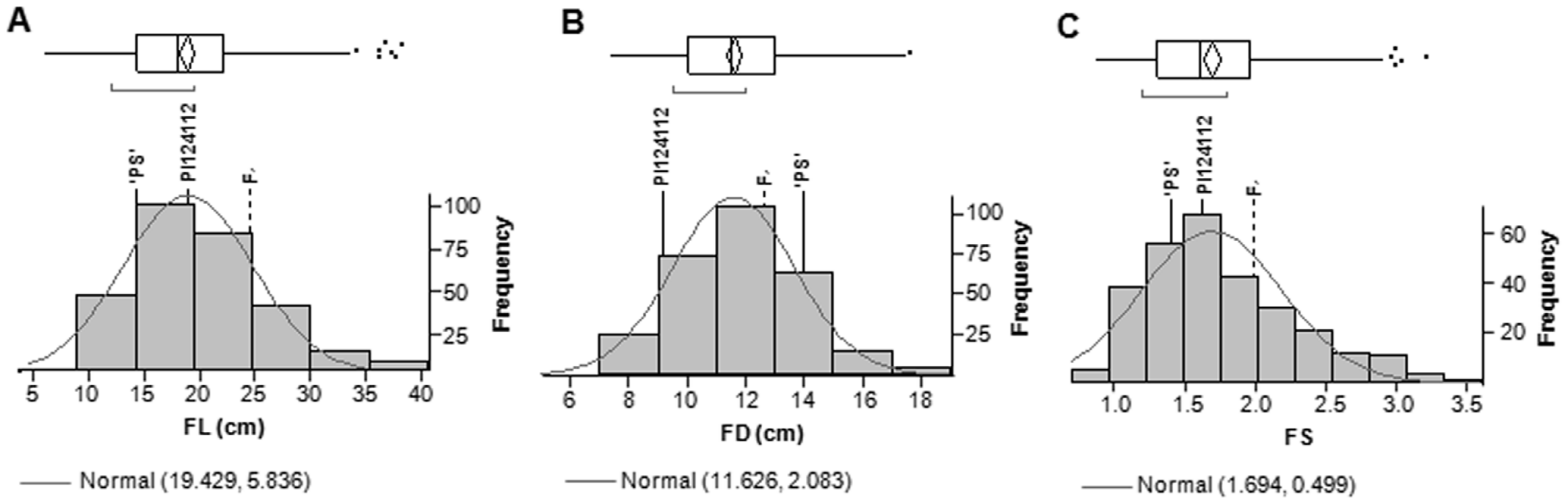
Frequency distribution of the traits across the F_2_ population derived from the cross ‘PS’ × PI124112. (A) FL (Fruit Length), (B) FD (Fruit Diameter) and (C) FS (Fruit Shape). Both parents and F_1_ values are marked. In the upper box, 25^th^, 50^th^ and 75^th^ quartiles are displayed; the sample mean and the 95% confidence interval are represented by a diamond, and the outlier values as dots. The bracket along the edge of the box stands for the part of the graph in which 50% of the observations are gathered together. Mean and standard deviation estimates for a normal distribution are also shown.

A continuous distribution of values was observed in the F_2_ (Figure 2), as expected in a quantitative trait like FS, with values ranging from 0.86 (oblate fruits) to 3.23 (extremely elongated fruits), making clear a transgressive segregation for this character in this population (Figure 1D and Figure 2C). Transgressive segregation was also observed for FL. In both cases, it was observed in both directions in the whole F_2_ population (Table 1 and Figure 2), although the phenotypes where more extreme for the higher values, that means fruits with a more elongated shape. In all cases, individuals with fruits exceeding the mid-parent value ±3.84SD were found. The trait showing a broader range of values was FL (from 7.1 to 38 cm), displaying extreme values corresponding to −6.56 and +13.52 times the SD of the mid-parental FL. Conversely, FD showed the lowest variation (from 7.30 to 17.70 cm), what represents the mid-parent FD value −3.84SD and +5.55SD, respectively. In between these two cases, the FS values ranged from −4.31SD and +11.23SD the mid-parent values, respectively.

FL and FS showed a highly significant (p<0.001) and strong correlation with values for the Pearson's correlation coefficient of 0.84 for the whole population, and 0.86 and 0.83 for the subpopulations of Cabrils and Zaragoza, respectively. In contrast, FD and FS were not significantly correlated in any case.

### Linkage map construction

**Figure 3:**
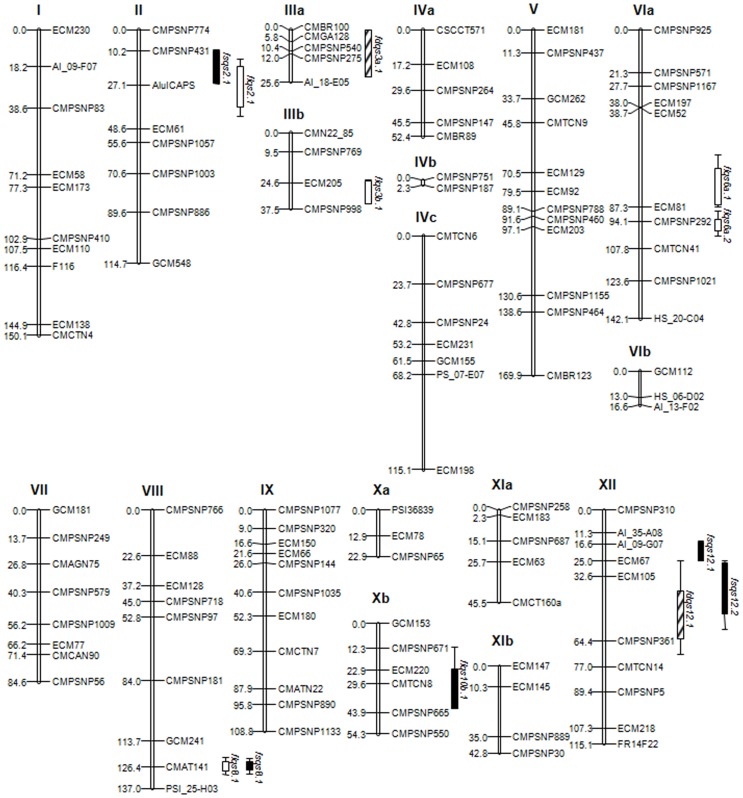
Location of 10 QTL responsible for morphological fruit traits mapped in a F_2_ population derived from the cross ‘PS’ × PI124112. FL: Fruit length, open bars; FD: Fruit Diameter, hatched bars; and FS: Fruit Shape, solid bars. Length of QTL bars corresponds to the two-LOD support intervals (from peak marker) based on the results obtained by CIM using Windows QTL Cartographer 2.5. Numbers on the left side correspond to the distance in cM (according to [44]) from the top of each chromosome.

One hundred and twenty-two out of the 133 total markers (63 SNPs, 57 SSRs and 2 CAPS) used to genotype the F_2_ mapping population could be incorporated to a genetic map consisting in 18 LGs that span 1437.3 cM (Figure 3), what renders a density of 1 marker every 11.8 cM. By comparing with the melon consensus map [48], each LG could be identified, finding that five of them were partial LGs of the integrated map. The map shows an average marker gap of 13.8 cM.

### QTL associated with fruit morphology traits

Given that no G×E interaction was found among locations, data from both locations were merged for subsequent QTL analysis. A total of ten QTL for fruit morphology-related traits were positioned along the melon genetic map (Table 2 and Figure 3) with the selective genotyping strategy, five underlying FL (in LGs II, IIIb, VIa, VIII and Xb), two responsible for FD (in LGs IIIa and XII), and three controlling FS (in LGs II, VIII and XII). LOD score values ranged from 2.80 to 16.85, in the case of *fdqs3a.1* and *flqs8.1*, respectively. The QTL with the highest effect for each of the three characters studied were *flqs8.1* (LOD = 16.85, R^2^ = 34%), *fdqs12.1* (LOD = 3.47, R^2^ = 11%) and *fsqs8.1* (LOD = 14.85, R^2^ = 41%). The remaining QTL accounted for 4 to 17% of the phenotypic variation. LOD peaks of the two major QTL located in LG II, *flqs2.1* and *fsqs2.1*, and in LG VIII, *flqs8.1* and *fsqs8.1*, overlapped completely (Figure S2).

**Table 2:** QTL detected for FL, FD and FS using the merged data from two locations of the F_2_ population from the cross ‘PS’ × PI124112.

Trait	LG	QTL name^a^	Flanking markers	Interval size (cM)	LOD score	*a* b	*d* b	d/[a]^b^	R^2c^	Homologue QTL ID^a^	Homologue published QTL^d^	Reference^e^
FL	II	*flqs2.1*	CMPSNP431-ECM61	38.4	8.44	3.06	2.29	0.75	0.16	*flqn2.1*	*fl2.1*	[54]
FL	IIIb	*flqs3b.1*	ECM205-CMPSNP998	12.9	2.92	+			0.05	*flqc3.5*	*3.5*	[14]
FL	VIa	*flqs6a.1*	ECM52-CMTCN41	69.1	9.56	+			0.11	*flqc6.4*	*6.4*	[14]
FL	VIII	*flqs8.1*	GCM241-PSI_25-H03	23.3	16.85	−4.95	−1.71	−0.35	0.34	*flqc8.3*	*8.3*	[14]
										*flqn8.1*	*fl8.1*	[54]
FL	Xb	*flqs10b.1*	CMPSNP671-CMPSNP665	31.6	3.02	–			0.04	–	–	–
FD	IIIa	*fdqs3a.1*	CMBR100-AI_18-E05	25.6	2.80	–			0.11	–	–	–
FD	XII	*fdqs12.1*	ECM67-CMTCN14	52	3.47	−1.03	−0.17	−0.17	0.11	*fdqi12.1*	*fw6.10*	[65]
FS	II	*fsqs2.1*	CMPSNP431-AluICAPS	16.9	6.74	0.27	0.20	0.75	0.17	*fsqj2.1*	*fs2.3*	[15]
										*fsqq2.1*	*fs2.2*	[12]
										*fsqn2.1*	*fsh2.1*	[54]
FS	VIII	*fsqs8.1*	GCM241-PSI_25-H03	23.3	14.85	−0.50	−0.16	−0.32	0.41	*fsqc8.3*	*8.1 in*	[14]
										*fsqn8.1*	*fsh8.1*	[54]
FS	XII	*fsqs12.1*	AI_35-A08-CMPSNP361	53.1	3.61	0.15	0.03	0.21	0.03	*fsqa12.1*	*fs11.1*	[13]
										*fsqp12.1*	*fs12.1*	[12]
										*fsqc12.1*	*12.1*	[14]

FL: Fruit Length; FD: Fruit Diameter; FS: Fruit Shape.

a Nomenclature according to [48].

b Only calculated for QTL in which the whole population was genotyped with the strongest linked marker(s); for the rest, only the sign is shown.

c When possible, calculated using the data coming the whole population genotyped with the strongest linked marker(s); for the rest, the value obtained by the QTL analysis using the selective genotyping subset of samples is shown.

d Nomenclature according to the authors that originally identified the QTL.

e Reviewed in [48] and, the FS QTL, in [53].

The interval defined by the markers flanking the QTL ranged from 12.9 (*flqs3b.1*) to 69.1 (*flqs6a.1*) cM, being less than 26 cM for five out of the ten QTL (Table 2).

The QTL on LG VIII (*flqs8.1* and *fsqs8.1*) were consistently detected in the three analyses (that is, with the merged data and with those coming from individual locations, File S3). The QTL *fsqs2.1* was also detected in the three analyses with a LOD = 2.5.The rests of the QTL were identified in two of the analyses, except for *flqs10b.1*. In all cases, the additive effects sign remained invariable across the different environments.

Since *fsqs2.1*, *fsqs8.1* and *fsqs12.1* were the QTL most consistently detected among analyses, all plants were genotyped with markers AluICAPS, CMAT141, and ECM67, located in the respective QTL region, in order to obtain unbiased estimates of their effects (Table 2). Same direction effects of the exotic allele (PI124112) for FL and FS QTL with the same chromosomal location were observed. In this sense, *flqs2.1* and *fsqs2.1* displayed a positive value for the additive effect (*a*), 3.06 and 0.27, respectively, what means that the PI124112 allele causes an increase in FL and FS, producing more elongated fruits. Conversely, the negative value of *a* for *flqs8.1* and *fsqs8.1* (−4.95 and −0.50, respectively) is indicative of a decrease in both characters, FL and FS, what results in rounder fruits, even if PI124112 itself produces very elongated melons. In all the remaining QTL, except for *fdqs12.1*, detected with the data coming from Cabrils and with the whole set (*a* = −1.03 and *a* = −1.10, respectively), the PI124112 allele contributed to increase the value of the particular morphological trait (Table 2 and File S3). So, the PI124122 allele at *fdqs12.1* will render elongated fruits by decreasing their diameters, whereas in the case of *flqs2.1* and *fsqs2.1*, a similar phenotype will be obtained by increasing the length of the fruit.

Gene action values ranged from −0.35 and −0.32 (*flqs8.1* and *fsqs8.1*, respectively) to 0.75 (*flqs2.1* and *fsqs2.1*; Table 2). In most cases, the alleles at the QTL *loci* showed an additive effect, except for *flqs2.1* and *fsqs2.1*, where the inheritance mode is near complete dominance. In general, the gene action values obtained for the same QTL using the subsets of data coming from both locations (File S3) agree with the ones calculated when all the data were pooled together, with slight differences that can be attributed to the smaller sample size of the first analysis.

The additive effect at *fsqs8.1* is supported by the phenotypic data, as the heterozygous NIL-*fsqs8.1* displays a FS value (i.e. 1.24) intermediate between the two homozygotes, the homozygous line for the PI124112 allele (i.e. 1.06) and the ‘PS’ parent (average FS = 1.40).

### QTL × QTL interaction

**Figure 4:**
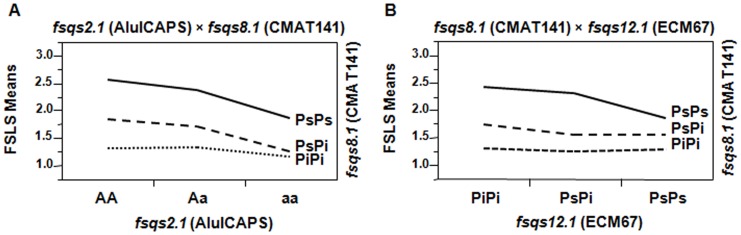
Digenic interactions studied by two-way ANOVA between the FS QTL using the markers significantly linked to them. (A) *fsqs8.1* and *fsqs2.1* (*fsqs2.1* × *fsqs8.1*); (B) *fsqs12.1* (*fsqs8.1* × *fsqs12.1*). Alleles at *locus flqs2.1* are named A and a since this QTL has been previously identified as gene *a* [12], [49]. PsPs: homozygous for the allele PS (solid line); PsPi: heterozygous (dashed line); PiPi: homozygous for the allele PI124112 (dotted line).

In order to detect the putative effects of other genomic regions on each of the FS QTL, all the possible digenic interactions between them were studied although only those that were statistically significant when *p*≤0.05 are shown (Table 3 and Figure 4).

**Table 3:** Significant digenic interactions between all the FS QTL.

QTL pair	Source	DF	Sum of Squares	F Ratio	Prob> F
*fsqs2.1*-*fsqs8.1*	*fsqs2.1* (AluICAPS)	2	7.83	35.72	<0.0001
	*fsqs8.1* (CMAT141)	2	26.79	122.21	<0.0001
	*fsqs2.1* (AluICAPS) × *fsqs8.1* (CMAT141)	4	1.42	3.24	0.0132*
*fsqs8.1*-*fsqs12.1*	*fsqs8.1* (CMAT141)	2	23.02	82.76	<0.0001
	*fsqs12.1* (ECM67)	2	1.59	5.71	0.0038
	*fsqs8.1* (CMAT141) × *fsqs12.1* (ECM67)	4	1.78	3.19	0.0142*

The interactions were explored by two-way ANOVA using the genotypes for the closest markers in the whole F_2_ population derived from the cross ‘PS’ × PI124112.

^*^: Interactions were considered to be statistically significant when *p*≤0.05.

### fsqs2.1 × fsqs8.1

A significant interaction was identified between *fsqs2.1* and *fsqs8.1* (p = 0.013, Figure 4A, Table 3), that explains a 4% of the genetic variance of FS.

*flqs2.1* has been previously identified as gene *a* [12], [49] and the marker linked to it (AluICAPS) is actually the causal mutation of andromonoecy [11]. For that reason and from now on, the alleles at this *locus* will be referred to as A and a.

It is well known that the dominant allele A causes elongated shapes in some genetic backgrounds [12], as becomes clear in the cases of homozygous for PS allele at locus *fsqs8.1* (PsPs). For each of the three possible genotypes at *locus a* (AA, Aa, aa), the lowest values of FS index (rounder fruits) were always observed in the homozygous for the PI124112 allele at locus *fsqs8.1* (PiPi) (Figure 4A). So, when the PI124112 allele is present at *fsqs8.1* and the dominant A allele is absent at locus *a* (PsPiaa and PiPiaa), there is a considerable decrease of FS.

In the homozygous PiPi, the differences among the three different genotypes at locus *a* are the slightest. It seems that homozygous PiPi at *fsqs8.1* diminish or, in some way, attenuates the effects of the dominant A allele of gene *a* on fruit elongation.

In the case of heterozygous genotypes at *fsqs8.1* (PsPi), some differences can be observed depending on the alleles present at locus *a* (A_ are expected to produce moderately elongated fruits while aa will rendered more rounded ones). So, we can only see marked differences in the three possible genotypes at gene *a* when the PI124112 allele is not present at QTL *fsqs8.1* (no masking effect) or it is in heterozygosis, PsPs or PsPi (moderate attenuation of the phenotype caused by A), with FS showing nearly parallel increments when these two scenarios are compared. In both cases, the elongated phenotype caused by the aforementioned dominant A allele becomes clear, as homozygous AA produce the most elongated fruits, followed by the heterozygous (Aa).

### fsqs8.1 × fsqs12.1

A significant interaction was identified between *fsqs8.1* and *fsqs12.1* (p = 0.014, Figure 4B, table 3), explaining 7% of the genetic variance of FS.

Similarly to what can be observed in the interaction *fsqs2.1 × fsqs8.1*, the lowest values of FS (rounder fruits) are obtained when the PI124112 allele is in homozygosis at *fsqs8.1* QTL (PiPi) (Figure 4B). In such a case, the phenotypic variations due to the different allelic composition at *fsqs12.1 locus* are practically imperceptible. Those differences in the FS value become more evident with the presence of the PS allele at *fsqs8.1*, specifically when it is in homozygosis (PsPs). Interestingly, the highest value of FS (the most elongated fruits) are reached for the PS homozygous at *fsqs8.1 locus* that are homozygous for the PI124112 at *fsqs12.1* (PsPsPiPi), what is totally in line with the positive additive value of *fsqs12.1* QTL (the Pi allele causes an increase in the FS index).

Here, unlike what happens in the previous interaction, within the same genotype for QTL *fsqs8.1*, the increase in FS due to the PI124112 allele at *fsqs12.1* becomes clear when the PI124112 allele is absent at locus *fsqs8.1*.

### QTL validation in the fsqs8.1-NIL

The identification and mapping of the *fsqs8.1* QTL in the F_2_ population has allowed us to use marker-assisted selection to precisely introgress the PI124112 allele responsible for the round shape into the elite cultivar ‘PS’ as the result of a backcross program (Figure S1). To ensure the integrity and maintenance of the QTL effects, two rounds of QTL verification were carried out in intermediate generations.

In 2008, the effect of the *fsqs8.1* was monitored with markers CMAT141 and PSI_25-H03 in BC4 plants (Table 4, 8M-codes in Figure S1). Plants carrying the PI124112 allele in heterozygosis at *fsqs8.1* produced significantly rounder melons than plants carrying the PS allele in homozygosis (F = 21.10 and F = 25.94 for CMAT141 and PS_25-H03, respectively, p<0.001).

**Table 4:** F-values obtained from ANOVA to validate the effects of QTL *fsqs8.1* on FS.

	2008		2009		2012
	CMAT141	PSI_25-H03	CMAT141	PSI_25-H03	CMAT141
FL	5.58^*^	8.76^**^	10.10***	4.82^*^	10.27***
FD	3.46^ns^	2.49^ns^	0.53^ns^	0.38^ns^	10.90^**^
FS	21.10***	25.94***	13.75***	5.71^**^	105.71***

Data coming from the assays carried out along 3 years were used. Plants were genotyped with linked markers (CMAT141 and/or PSI_25-H03).

FL: Fruit Length; FD: Fruit Diameter; FS: Fruit Shape.

^***^P<0.001;^**^P<0.01; ^*^P<0.05; ^ns^P>0.05.

In 2009, the three genotypes at *fsqs8.1* (monitored with marker CMAT141 and PSI_25-H03) segregated in a BC4S1 population (9M7, Figure S1), re-confirming the cosegregation of PI124112 alleles at *fsqs8.1* with the round fruit phenotype (Table 4; F = 13.75 and p<0.001 for CMAT141; F = 5.71 and p<0.01 for PSI_25-H03).

At the next stage, whole-genome genotyping was performed in a selected BC5 plant (10M2-30) in order to check the integrity of the introgressed region and the composition of the genetic background. The target introgression harbored 8.5 Mb in the LG VIII, and there were only seven additional introgressions ranging from 250 kb to 86.8 Mb.

Finally, the definitive introgression line was fixed in a BC5S2 family (12M57, Figure S1). The family 12M57 showed highly significant shorter and rounder fruits, that is, lower values of FL and FS, respectively (Table 4; F = 10.27 and F = 105.71, for FL and FS, respectively, and p<0.001). Additionally, significant effects on FD were also observed (F = 10.90, p<0.001, Table 4).

## Discussion

The F_2_ population between ‘PS’ and PI124112 showed an impressive variability for fruit morphology. Transgressive segregation in both directions was found for FL and FS, indicating that at least one of the parents contributed alleles with opposite effects and making possible to obtain genotypes that could render round fruits. In fact, the existence of an allele inducing round-shaped melons was identified in the genitor PI124112 and verified with an introgression line in the ‘PS’ background. The effects of this allele seem to be masked by epistatic interactions with other gene(s) in the PI124112 background, and those effects were unveiled in the ‘PS’ background. A similar discovery has been reported recently with the obtaining of a climacteric line derived from the cross between two non-climateric melon accessions, ‘PS’ and ‘Shongwan Charmi’ [37]. This is a further indication of the richness of the melon gene pool that remains still largely unexplored.

The segregating population coming from parents genetically distant and with morphological differences used in this work has revealed itself as suitable for the genetic dissection of morphological traits and mapping QTL. Furthermore, the most robust FS (and the highly correlated FL) QTL detected (*flqs2.1*, *fsqs2.1*, *flqs8.1* and *fsqs8.1*) were consistent across the locations tested, in agreement with other results published [16], in which QTL affecting FS displayed a high heritability, what explained their reproducibility in assays carried out in different locations and along several years.

The availability of a melon consensus map including the position of QTL controlling a panoply of important traits [48] made easier the comparison of the QTL identified here with those previously described. In this sense, most QTL controlling the same characters studied in this work have been detected previously and mapped to similar genomic positions (Table 2). In fact, homologous QTL obtained in different populations can be assigned to all the QTL described here, except in the case of *flqs10b.1* (only detected with the full set of data) and *fdqs3a.1* (not found in Cabrils). Only a QTL associated to FL was recently reported in the LG II of the related cucurbit Zucchini (*Cucurbita pepo* L.) in a region syntenic to the LG III of melon at which *fdqs3a.1* maps [50]. However, this QTL could not be confirmed in further backcross generations.

The QTL in LG II (*flqs2.1* and/or *fsqs2.1*) was previously identified as a pleiotropic effect of the gene *a* [12], [49], whose alleles are responsible for sex determination in melon flowers [51] as it has been reported in other plant species like *Vitis* ssp. [52]. The dominant inheritance mode observed for the alleles at *flqs2.1* and *fsqs2.1 loci* also supports it. The cloning of the gene has unveiled that the causal mutation of the andromonoecious melon types is in the active site of 1-aminocyclopropane-1-carboxylic acid synthase [11].

The map position of *fsqs8.1* (and *flqs8.1*, as they overlap) happened to be similar to those reported for other FS QTL (Table 2; reviewed in [48], [53]), *fsqc8.3* (*8.1* in [14]) and *fsqn8.1* (*fsh8.1* in [54]). Those QTL were detected in a NIL population derived from the cross ‘PS’ (*C. melo* var. *inodurus*) × PI161375 ‘Shongwan Charmi’ (*C.* melo var. *chinensis*), and a Recombinant Inbred Line (RIL) population coming from the cross PI414723 (*C. melo* var. *momordica*) × ‘Dulce’ (*C. melo* var. *reticulatus*), respectively. So, *fsqs8.1* seems to be allelic to those major QTL underlying fruit morphology in a broad array of melon germplasm. The PI161375 and PI414723 alleles produced elongated fruits. On the contrary, PI124112 allele (in a certain allelic combination in *trans* with gene *a* variants) induced round fruits, what may be showing the existence of an allelic series at this QTL when different germplasm sources are investigated. Three more QTL have been reported in the surrounding region (*fsqa8.1*, *fsqn8.1* and *fsqc8.1*, compiled in [48]) and two additional ones further away in the same chromosome (*fsqc8.1* and *fsqp8.2*, reviewed in [48]). These results suggest that an important part of the *loci* controlling FS in melon could be concentrated in the LG VIII. It is also possible that some of them are the same QTL that the one described here (harboring common and/or new alleles). To discern between these two cases, higher resolution in the mapping and QTL studies is required. Similar QTL clusters have also been observed for other traits in tomato [55] and maize [56] – [57]. Furthermore, all the QTL for FL, which is strongly correlated to FS (the authors didn't consider the FS ratio as a trait), identified by [58] in cucumber (another member of the genus *Cucumis*), were located in LG 4, what is syntenic to LG VIII of *C. melo* together with LG 6 of cucumber [59]–[60]. In agreement with this, most of the FS QTL found by [61] were located in cucumber in those two same chromosomes, 4 and 6. In Zucchini, however, the reported major QTL involved in fruit shape was mapped in LG VI, which is syntenic to LG IV of melons and Chr3 of cucumber [50].

If most of the fruit morphological variability present in cultivated melons is attributable to an array of alleles at a limited number of *loci*, as it seems happens in tomato [62]), then their unambiguous characterization provides scientists and breeders with an useful tool to engineer it.

The FS QTL *fsqs12.1* identified in this work maps in the neighborhood of the chromosomal segment harboring the gene *p*, which controls carpel number. Pleiotropic effects of *p* on fruit shape has been reported previously [12]–[13], similarly to what happens with the gene *a*. Also, fruit shape variation has been related to differences in the number of carpels in *C. pepo*. A QTL associated to this variation has been mapped in LG V in a region of *C.pepo* genome that is syntenic to that one in melon LG XII in which the *p* gene is located [50].

In a series of NILs derived from ‘PS’ (produces melons with three carpels) and the accession ‘Shongwan Charmi’ (renders fruits with five carpels), fruits with five carpels were rounder than those with three [14], [16]–[17]. However, a high number of carpels is an undesirable feature in commercial types as it causes the fruit to have a bigger empty cavity. The carpel number did not segregate in the population studied in this work, therefore the variation in the number of carpels is not a consequence of *fsqs12.1* effects. On the other hand, it is also possible that at least part of the fruit morphology variation associated to *p* in the other populations could be caused by another linked FS QTL. The *fsqc12.1* QTL was mapped within a 5 cM interval by [17], further genetic resolution would be necessary to discard the presence of another linked QTL.

When QTL responsible for FS identified in tomato, eggplant and pepper have been compared, an interesting scenario is revealed, as they could be orthologous *loci* controlling the same character in all these species. In line with this, the same connection perhaps can be established with melon. To do it, the recently obtained melon genome sequence [60] would be of great value.

Positive values of the additive effect of QTL in LG II, *flqs2.1* and *fsqs2.1* (Table 2), indicate that alleles from PI124112 act in the direction of increasing the magnitude of both FL and FS. The same can be argued for *fsqs12.1* QTL regarding FS. Surprisingly, it is quite the opposite for the QTL in LG VIII, *flqs8.1* and *fsqs8.1*, which display negative additive effects (Table 2). So, for this QTL, the exotic PI124112 alleles contribute to decrease the values of FL and FS, rendering rounder fruits even if PI124112 itself produces very elongated melons [5]. Furthermore, it is well known that the dominant alleles of gene *a* (A), responsible for the monoecious sex type (A_), also causes elongated melon fruits in most genetic backgrounds. However, andromonoecious cultivars (aa) that produce elongated fruits are also common (i.e. ‘PS’). Evidence for very significant interactions between these two QTL, *fsqs2.1* and *fsqs8.1* (Table 3 and Figure 4A), may shed light on this. So, if no interaction would exist, round fruits would be only produced by homozygous for the exotic allele at *fsqs8.1* that do not carry any dominant A allele at locus *a* (PiPiaa). It seems that there is a requisite that must be satisfied in order to obtain a round fruit and that is to have two copies of the PI124112 allele at the *fsqs8.1* locus. Therefore, only the homozygous (PiPi) can potentially render rounded melons. Furthermore, for these homozygous PiPi, the presence of the A allele at gene *a* has practically no effect on the elongation of the fruit and it is then when the epistasis between these two *loci* becomes obvious. Only in this scenario, the real effect of the PI124112 alleles at *fsqs8.1* is clear. Among the heterozygous at *fsqs8.1*, only those not having any A allele at locus *a* (PsPiaa) will be able to produce moderately elongated fruits. The rest (PsPiAA and PsPiAa), will give very elongated fruits. According to this, the homozygous PsPsAA generates the most elongated fruits, followed by PsPsAa.

The additive effect of alleles at *fsqs8.1* has been confirmed by the phenotype of the heterozygous NIL-*fsqs8.1*, with a FS value intermediate between the two homozygotes. This agrees with the results observed by [16] in a population derived from the cross ‘PS’ × ‘Shongwan Charmi’, in which the QTL alleles inducing fruit enlargement were dominant, whereas those inducing rounder fruit were additive or recessive and only minor epistatic interactions were found.

So, the action of the QTL *fsqs8.1* attenuates the effects of the alleles at locus *a*. Therefore, the differences due to the genotype at the latter are more evident when the PI124112 allele is absent at *fsqs8.1* (PsPs, in our case), as it has been previously described in cases of QTL epistatic interactions in other fruits, like tomato [63].

Adding complexity to this scenario, the other interaction detected between two FS QTL, *fsqs8.1* and *fsqs12.1*, shows a similar pattern though, in this case, the exotic allele at *fsqs12.1* causes the enlargement of the fruit. This means that the roundest fruits will be produced by individuals that are homozygous for the PI124112 allele at *fsqs8.1* and for the ‘PS’ allele at *fsqs12.2* (PiPiPsPs). On the other hand, the most elongated fruits will be obtained from the other possible double homozygous (PsPsPiPi). In between, the heterozygous will show a wide range of FS values, with the double heterozygous exhibiting an intermediate phenotype.

To conclude, the effect of the PI124112 allele for *fsqs8.1* remains unnoticed in the PI124112 accession. Now, this becomes understandable because alleles for FS QTL with opposite effects have been detected in the accession PI124112. To unveil the interactions reported here has been possible thank to the isolation of the effect of *fsqs8.1* from the interference of other FS QTL in the *fsqs8.1*-NIL.

The literature is riddle with examples of epistatic interactions among QTL affecting the same character, like *ljfs2* (probably corresponding to *ovate*), *ljfs7* (likely being *sun* and having pleiotropic effects on pericarp elongation), *ljfs3* (it maps close to *fs3.2)* and *jfs11* (in the same region that *fs11.1*), all of them affecting a morphological trait like eccentricity in tomato [64]. A strategy successfully employed to uncover QTL hidden by epistatic interactions with other genes has been to cross a certain QTL-IL (introgression line) with different genotypes, so new phenotypes arise in certain genetic backgrounds [63].

## Conclusions

The additive inheritance mode of *fsqs8.1*, with the exotic allele coming from PI124112 being the one causing the round shape, make the *fsqs8.1*-NIL a more suitable material to modify the morphology of commercial varieties by obtaining hybrids. Actually, monoic cultivars normally produce elongated fruits and for that reason, they are not commonly used to generate new commercial hybrids as the FL and FS values increase as a result of heterosis [5], [16]. However, monoecy brings some advantages from a breeding perspective since it eliminates the need for hand emasculation during the crossing process, and specifically to obtain hybrids, that usually produce fruits with improved quality traits. Therefore, unveiling and understanding epistatic relationships between the QTL affecting FS and gene *a*, like the one discovered with *fsqs8.1*, could make possible the development of hybrids that bring together both desirable characteristics, that is, potential new monoecious varieties producing round-shaped fruits.

## Supporting Information

Figure S1 Pedigree showing the time scale and the crosses carried out to generate the mapping F_2_ population and to eventually obtain the *fsqs8.1*-NIL.
(TIF)

Figure S2 Locations of QTL for FL (Fruit Length) and FS (Fruit Shape), *flqs2.1* and *fsqs2.1*, respectively, in chromosome II; and of *flqs8.1* and *fsqs8.1* on chromosome VIII, showing their overlapping. The threshold LOD (2.5) was calculated for a significant level p = 0.05 by a permutation test (n = 1000). Peak marker: in bold; flanking markers: underlined.
(TIF)

File S1 Morphological data in the 84 F_2_ individuals comprising the selective genotyping subpopulation for the three traits studied (FL, FD and FS).
(XLSX)

File S2 Markers used in the mapping and QTL verification experiments.
(XLSX)

File S3 QTL detected for FL (Fruit Length), FD (Fruit Diameter) and FS (Fruit Shape) using the merged data from two locations of the F_2_ population from the cross ‘PS’ × PI124112, and the two sets of data independently at a LOD calculated for a significant level p = 0.05 by a permutation test (n = 1000) and at LOD>2.5.
(XLSX)
