# Correction: Mapping and Introgression of QTL Involved in Fruit Shape Transgressive Segregation into ‘Piel de Sapo’ Melon (*Cucucumis melo* L.)

**DOI:** 10.1371/journal.pone.0116098

**Published:** 2014-12-19

**Authors:** The PLOS ONE Staff

The species name is misspelled in the article title. The correct title is: Mapping and Introgression of QTL Involved in Fruit Shape Transgressive Segregation into ‘Piel de Sapo’ Melon (*Cucumis melo* L.). The correct citation is: Díaz A, Zarouri B, Fergany M, Eduardo I, Álvarez JM, et al. (2014) Mapping and Introgression of QTL Involved in Fruit Shape Transgressive Segregation into ‘Piel de Sapo’ Melon (*Cucumis melo* L.). PLoS ONE 9(8): e104188. doi:10.1371/journal.pone.0104188
