# Supplementary figures and images for: Mapping and Introgression of QTL Involved in Fruit Shape Transgressive Segregation into ‘Piel de Sapo’ Melon (Cucucumis melo L.)
Source: PLoS One. 2014 Aug 15;9(8):e104188. doi: 10.1371/journal.pone.0104188 (PMC4134209; doi:10.1371/journal.pone.0104188)

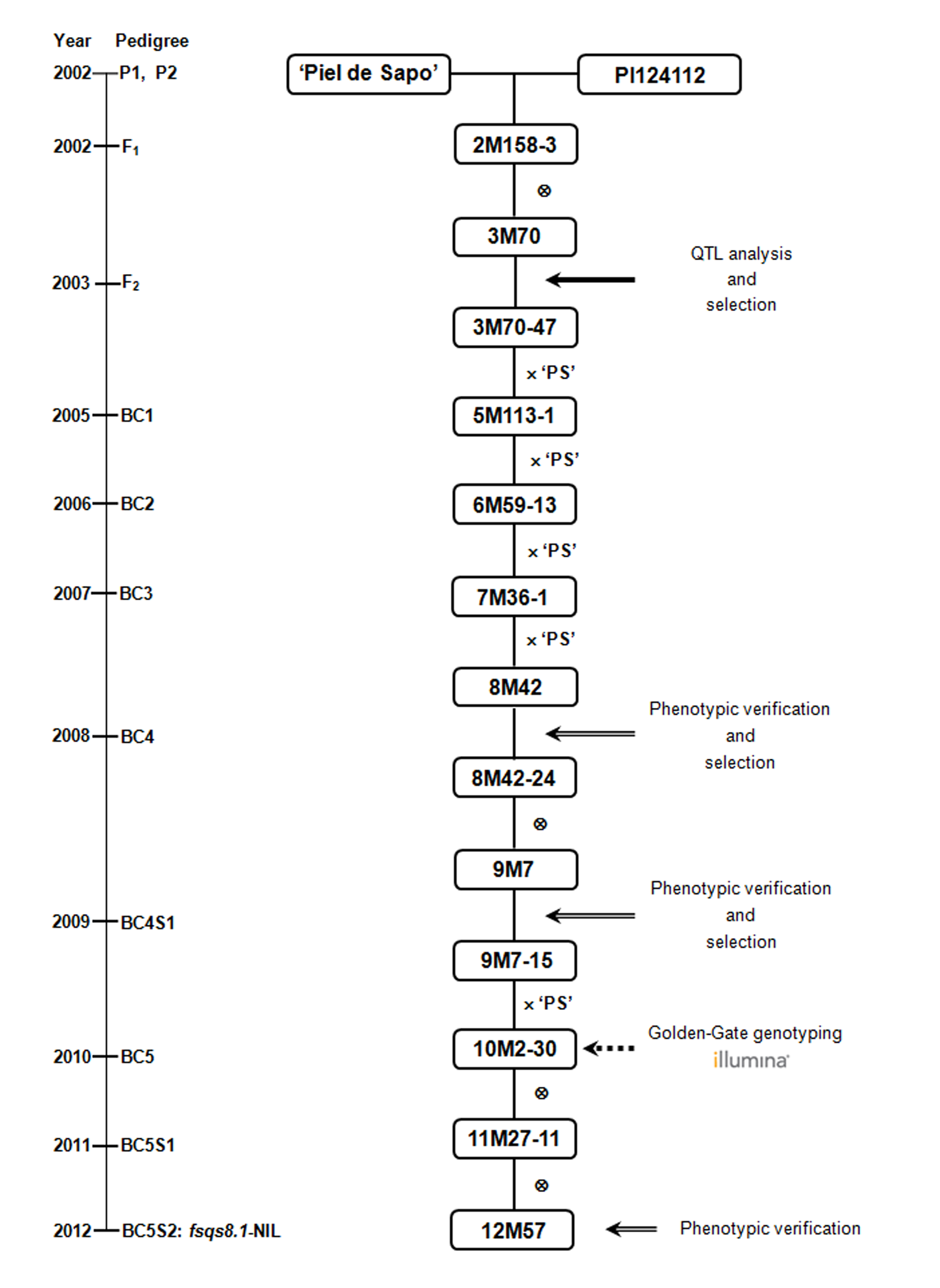

Supplement: Figure S1 — Pedigree showing the time scale and the crosses carried out to generate the mapping F2 population and to eventually obtain the fsqs8.1-NIL. (TIF) [file pone.0104188.s001.tif]

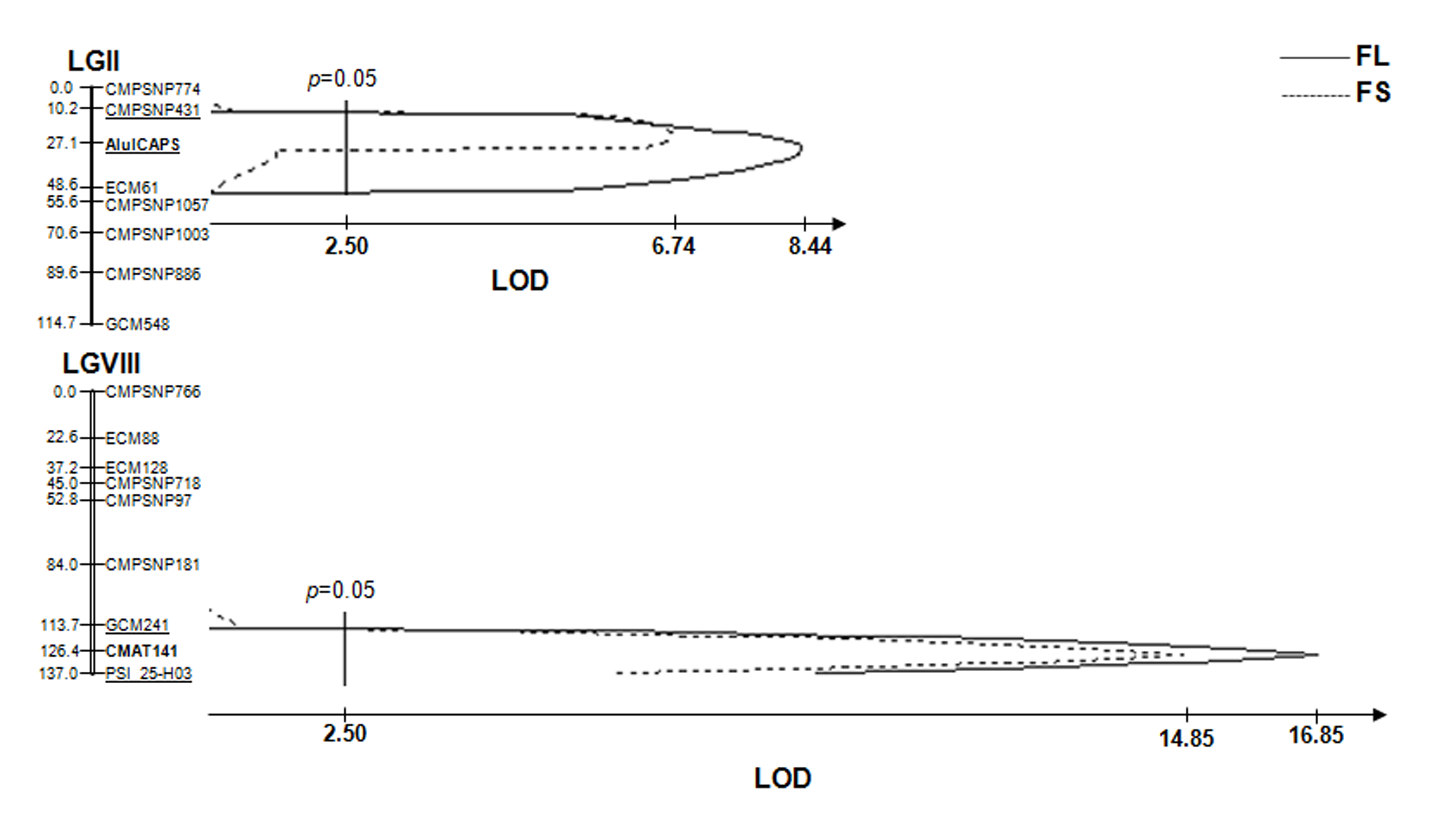

Supplement: Figure S2 — Locations of QTL for FL (Fruit Length) and FS (Fruit Shape), flqs2.1 and fsqs2.1, respectively, in chromosome II; and of flqs8.1 and fsqs8.1 on chromosome VIII, showing their overlapping. The threshold LOD (2.5) was calculated for a significant level p = 0.05 by a permutation test (n = 1000). Peak marker: in bold; flanking markers: underlined. (TIF) [file pone.0104188.s002.tif]
